# The anti-breast cancer property of physcion via oxidative stress-mediated mitochondrial apoptosis and immune response

**DOI:** 10.1080/13880209.2021.1889002

**Published:** 2021-03-14

**Authors:** Luping Zhang, Ruitao Dong, Yu Wang, Longxiang Wang, Tian Zhou, Dongxu Jia, Zhaoli Meng

**Affiliations:** a The Gastroenterology & Endoscopy Center, First Hospital, Jilin University, Changchun, Jilin, China; b School of Life Sciences, Jilin University, Changchun, China; c Department of Translational Medicine Research Institute, First Hospital, Jilin University, Changchun, Jilin, China

**Keywords:** Breast cancer, nuclear factor-kappa B, nuclear factor erythroid 2-related factor 2, cytotoxicity

## Abstract

**Context:**

Physcion (Phy) exerts several pharmacological effects including anti-inflammatory, antioxidant, and antitumor properties.

**Objective:**

This study investigates the cytotoxicity and its underlying mechanisms of Phy on breast cancer.

**Materials and methods:**

Human breast cancer cell MCF-7 was treated with 5–400 µM Phy for 24 h, MCF-7-xenografted BALB/c nude mice and immunosuppressive mice model induced by cyclophosphamide were intraperitoneally injected with 0.1 mL/mouse normal saline (control group) and 30 mg/kg Phy every other day for 14 or 28 days, and pathological examination, ELISA and western blot were employed to investigate the Phy anti-breast cancer property *in vitro* and *in vivo*.

**Results:**

In MCF-7 cells, Phy 24 h treatment significantly reduced the cell viability at dose of 50–400 µM and 24 h, with an IC_50_ of 203.1 µM, and 200 µM Phy induced 56.9, 46.9, 36.9, and 46.9% increment on LDH and caspase-3, −8 and −9. In MCF-7-xenograft tumour nude mice and immunosuppressive mice, 30 mg/kg Phy treatment inhibited tumour growth from the 8th day, and reduced Bcl-2 and Bcl-xL >50%, HO-1 and SOD-1 > 70% in tumour tissues of immunosuppressive mice. In addition, Phy reduced nuclear factor erythroid 2-related factor 2 > 30% and its downstream proteins, and enhanced the phosphorylation of nuclear factor-kappa B > 110% and inhibitor of NF-кB α > 80% in the tumour tissues of BALB/c mice.

**Discussion and conclusions:**

This research demonstrated that Phy has an anti-breast cancer property via the modulation of oxidative stress-mediated mitochondrial apoptosis and immune response, which provides a scientific basis for further research on its clinical applications.

## Introduction

As one of the most common malignancies in women, breast cancer induces changes in breast lump, nipple, and areola as clinical features, it remains the leading cause of cancer death among women in less developed countries (Torre et al. 2015). In modern society, the treatment of breast cancer has entered an era of multidisciplinary comprehensive treatment guided by biological characteristics, including molecular targeted therapy, endocrine therapy, chemotherapy, radiotherapy, and surgery. Although progress has been made in the treatment of breast cancer, surgical treatment is still an indispensable part of comprehensive treatment for international stage II and stage III patients which usually makes breast cancer patients prone to severe psychological disorders and even causes anxiety or depression.

Therefore, there is an urgent need for more effective therapeutic agents for breast cancer treatment. Due to the expensive and various adverse effects of chemically synthesized agents, modern scientists are turning their attention to green natural medicines such as plants and minerals or their derivatives. Among them, pyhscion (Phy) extracted from *Rheum officinale* Baill. (Polygonaceae) is a naturally occurring anthraquinone derivative with various biological activities including antimicrobial, antiviral, anti-inflammatory, protecting cerebral cortical neurons, repairing damaged DNA, and antitumor (Pan et al. 2019). Phy and its derivatives have demonstrated antitumor activities via inducing apoptosis, decreasing metastasis and disrupting of cell cycle against various cancer cells such as hepatic carcinoma, lung cancer, nasopharyngeal carcinoma, prostate cancer, ovarian cancer and breast cancer (Xue et al. 2019; Xun et al. 2019). However, there are no reliable studies related to the antitumor activities of Phy on human breast cancer cell lines.

Known as programmed cell death, apoptosis effects in tumour formation and development primarily via the death receptor pathway and/or mitochondrial pathway (Elmore 2007; Pilipenko et al. 2019). Cysteinyl aspartate specific proteinase (caspase), a group of proteases with similar structure in the cytoplasm, is responsible for eukaryotic cell apoptosis (Green and Reed 1998). Caspase-8 and caspase-9 converge on the same execution pathway by activating caspase-3 leading to DNA fragmentation and formation of apoptotic bodies (Schultz and Harrington 2003). Mitochondria are the centre of cell respiratory chain, oxidative phosphorylation and apoptosis regulation (Desagher and Martinou 2000). The altered cellular oxidation-reduction, lost mitochondrial transmembrane potential, and the release of caspase activators are key steps in mitochondrial pathway (Green and Reed 1998). Bcl-2 family proteins are involved in apoptosis via altering mitochondrial membrane permeability (MMP), thereby regulating the release of cytochrome C, as well as the activation of downstream caspase-3 (Ow et al. 2008).

The development and metastasis of tumours is associated with immune regulation, inflammation and oxidative stress (DeNardo and Coussens 2007; Gill et al. 2016; Kudryavtseva et al. 2016). Oxidative stress causes the damage and modification of genomic DNA and other cellular macromolecules via activating or inhibiting the transcription factors and second messengers such as nuclear factor-kappa B (NF-кB), which is involved in the transcription of genes related to the immune response and inflammatory response related to the regulation on the expression of matrix metalloproteinase (MMPs) and interleukins (ILs) (Haddad 2004; Karin and Greten 2005; Taniguchi and Karin 2018). During the cellular oxidative stress responses, known as the transcription factor, nuclear factor E2-related factor (Nrf2) can stimulate its download gene expressions such as haem oxygenase-1 (HO-1), catalase (CAT), and superoxide dismutase (SOD) (Done and Traustadottir 2016).

In this study, the pro-apoptotic effects of Phy were successfully found in MCF-7 cells and in MCF-7-xenografted BALB/c nude mice. Additionally, in MCF-7-xenografted BALB/c mice, the anti-breast cancer property of Phy was confirmed via its modulation on oxidative stress-mediated immune response. Our research suggests the potential application value of Phy on the therapy of breast cancer.

## Materials and methods

### Materials

Physcion (Phy) (ASB-00016790-025), lactate dehydrogenase (LDH) cytotoxicity test kit (R24020) and mitochondrial membrane potential detection kit (R20230) were obtained from Shanghai Yuanye Bio-Technology Co., Ltd. (Shanghai, China). Annexin V-FITC apoptosis assay kit (4830-01-K) was obtained from BD Biosciences. Caspase-3 (G015-1-3), −8 (G017-1-3) and −9 (G018-1-3) activity assay kit were obtained from Nanjing Jiancheng Bioengineering Institute (Nanjing, China). Enzyme-linked immuno sorbent assay (ELISA) kits of IL-1β (KET7005), IL-2 (KET7006), IL-6 (KET7009), IL-10 (KET7010), tumour necrosis factor-α (TNF-α) (KET7015) were obtained from Abbkine Scientific Co., Ltd. (Wuhan, China). ELISA kits of TNF-β (MBS494101), matrix metalloproteinase-2 (MMP-2) (MMP200), MMP-9 (OKBB00227) and interferon-γ (IFN-γ) (ELM-IFNg-1) were purchased from Shanghai Yubo Biotechnology Co., Ltd. (Shanghai, China). The colorimetric assay kits of reactive oxygen species (ROS) (E-BC-K138-F), SOD (E-BC-K019-M) and CAT (E-BC-K031-M) were purchased from Elabscience Biotechnology Co., Ltd (Wuhan, China). BCA Protein Assay Kit (BCA1-1KT) was purchase from Merck Millipore (Billerica, MA, USA). Antibodies for cleaved-caspase-3 (ab49822), B-cell lymphoma-2 (Bcl-2) (ab182858), B-cell lymphoma-extra large (Bcl-xL) (ab32370), Bcl-2-associated X protein (Bax) (ab32503), Nrf2 (ab92946), HO-1 (ab13248), SOD1 (ab13498), SOD2 (ab137037), phosphor (P)-NF-кB (ab28849), total (T)-NF-кB (ab32360), reduced glyceraldehyde-phosphate dehydrogenase (GAPDH) (ab8245), and HRP-linked secondary antibody (ab6939) were purchased from Abcam (Cambridge, UK), and P-IкBα (SAB4504445) and T-IкBα (I0505) were purchased from Merck Millipore.

### Cell culture

MCF-7 (CRL-3435™) [the American Type Culture Collection (ATCC)] were cultured in Dulbecco’s Modified Eagle Media (DMEM) (12491015, Gibco, Invitrogen, CA, USA) containing 1% penicillin and streptomycin (15070063, Gibco, Invitrogen, CA, USA), and 10% foetal bovine serum (Zhejiang Tian hang bio Polytron Technologies Inc, China) at 37 °C with 5% CO_2_. The cells were digested and subcultured when 80% confluence was reached.

### Cell viability detection

The effects of Phy on cell viability was detected using 3-(4,5-dimethylthiazolyl-2)-2,5-diphenyltetrazolium bromide (MTT) (M2128, Sigma Aldrich, MO, USA) assay. MCF-7 cells were seeded in 96-well plates at 2 × 10^4^ per well. The medium containing 5, 25, 50, 100, 200 and 400 µM of Phy was added to replace the original culture medium after 24 h. MTT (0.5 mg/mL) (10 μL) reagent was added into each well in 4 h before the end of the 24 h incubation in the dark, and the absorbance was measured at a wavelength of 490 nm after adding 100 µL of dimethyl sulfoxide with Microplate Reader (BioTek Instruments, Winooski, VT, USA).

### The activities of caspases and LDH levels detection

The caspase activities and LDH release in Phy-treated cells were carried out using the relevant assay kits. MCF-7 cells were plated into 6-well plates at 1 × 10^5^ cells per well, and then exposed to 100 or 200 µM Phy for 24 h. After the incubation with Phy, cells were lysed in the ice bath and the medium was collected for determination of LDH release. The activities of caspase-3, -8, -9 and the concentration of LDH were analyzed following with the manufacturer's protocols.

### Cell apoptosis analysis

Cell apoptosis were evaluated via Annexin V-FITC/PI (MCH100105, EMD Merck Millipore, Billerica, MA, USA) double staining. MCF-7 cells were plated in 6-well plates at 1 × 10^5^ cells per well and incubated overnight. After exposed to 100 or 200 µM of Phy for 24 h, cells were routinely collected and rinsed twice with pre-chilled phosphate buffer (PBS) followed by 400 μL of binding buffer. Annexin V-FITC dye (10 μL) was added in the dark. After incubation at 4 °C for 10 min, 5 μL of PI dye was added and cells were incubated at 4 °C for 10 min continuously. Muse^®^ Cell Analyser (Millipore, Billerica, MA, USA) was used to perform cell apoptosis.

### Assessment of MMP

MCF-7 cells were plated into 6-well plates at 1 × 10^5^ cells/well, and incubated with Phy at 100 or 200 µM of Phy for 24 h. Treated cells were stained with 2 μM of 5,5′,6,6′-tetrachloro-1,1′,3,3′-tetraethylbenzimidazolylcarbocyanine iodide (JC-1) (MAK160, Sigma Aldrich, MO, USA) for 15 min at 37 °C in darkness. After washes, a fluorescence microscope (Eclipse TE 2000-S, Nikon Corp., Tokyo, Japan) was applied to detect the changes on the red/green fluorescence.

### MCF-7-xenografted tumour model development and agent administration protocol

Female BALB/c nude mice (5-week-old) and female BALB/c mice (8-week-old), purchased from Chares River Experimental Animal Technical Co., Ltd. (Beijing, China), were housed in clear plastic cages and maintained on a 12 h light/dark cycle at 22 ± 1 °C with water and food available *ad libitum.* This research was approved by the Animal Ethics Committee of School of Life Sciences, Jilin University (2017SY0601-2).

For BALB/c nude mice, total 6 × 10^6^ MCF-7 cells were subcutaneously implanted below the right back near hind leg. Once the tumour volume reached to 80–100 mm^3^, mice were divided into two groups randomly including control group (CTRL) intraperitoneally injected with 0.1 mL/mouse normal saline (*n* = 3), and Phy-treated group intraperitoneally injected with 30 mg/kg of Phy (total volume 0.1 mL) (*n* = 3) every other day for 14 days, respectively. Using [length (mm) × width (mm)^2^ × 0.5] the tumour volume was calculated. The body weight and tumour size measured by vernier calliper were recorded every other day.

For BALB/c mice, 50 mg/kg of cyclophosphamide (239785, Sigma Aldrich, MO, USA) was intraperitoneally injected for 3 days continuously and once per week for four weeks. Total 6 × 10^7^ MCF-7 cells were subcutaneously implanted below the right back near hind leg. After 5 days, mice were divided into two groups randomly including control group (CTRL) intraperitoneally injected with 0.1 mL/mouse normal saline (*n* = 3), and Phy-treated group intraperitoneally injected with 30 mg/kg of Phy (total volume 0.1 mL) (*n* = 3) every other day for 28 days, respectively. The body weight was recorded every other day.

Blood was sampled from the caudal vein of all experimental mice 4 h after the last agent treatment, and then euthanasia was performed via carbon dioxide suction. Tumour tissues and organs including liver and spleen were collected.

### Immune cytokines and oxidative factors detection

The serum levels of cytokines of MCF-7-xenografted BALB/c mice including IL-1β, IL-2, IL-6, IL-10, MMP-2, MMP-9, TNF-α, TNF-β and IFN-γ were detected by ELISA kits and the serum levels of ROS, SOD and CAT of MCF-7-xenografted BALB/c mice were analyzed by colorimetric assay kits according to the manufactural protocols.

### Histopathological examination

The collected liver and spleen were fixed in 4% formaldehyde (10010018, Sinopharm Chemical Reagent Co., Ltd) for 48 h. The fixed tissue specimens were harvested, dehydrated, transparent, impregnated with wax, and embedded, sectioned into 5 μm thickness by microtome (Leica, Wetzlar, Germany), and then stained with eosin and haematoxylin (H&E) according to previous methodology (Zhang et al. 2017).

### Western blotting

MCF-7 cells were exposed to 100 or 200 µM for 24 h. Collected cells and tumour tissues obtained from the two xenografted tumour bearing mice were homogenized in cold radio-immunoprecipitation assay (RIPA) lysis buffer (20–188, Millipore, Billerica, MA, USA) containing 1% cocktail inhibitors (524625, Millipore, Billerica, MA, USA). The protein concentration of cell lysates and tissue lysates were measured by the BCA Protein Assay Kit. Proteins (40 μg) were electrophoresed and separated with 10–12% sodium dodecyl sulphate polyacrylamide gel electrophoresis gel, and then transferred onto a polyvinylidene fluoride membranes (0.45 μm) (Millipore, Billerica, MA, USA), which was blocked with 5% bull serum albumin. After washes, the membranes were incubated with the primary antibodies including Bcl-2, Bax, Bcl-xL, Nrf2, HO-1, SOD1, SOD-2, p-NF-κB, NF-κB, p-IкBα, IкBα, and GAPDH overnight at 4 °C following with exposure to secondary antibody at 25 °C for 2 h. Protein bands was visualized by the Electro Chemi Luminescence (ECL) detection kits (WBULS0500, Merck Millipore, Billerica, MA, USA), and their intensity were quantified with Image J software (NIH, Bethesda, MD, USA).

### Statistical analysis

Data were presented as means ± S.D. and analyzed with one-way ANOVA, followed by Dunnett’s test for multiple comparisons using SPSS 16.0 software (IBM Corporation, Armonk, NY, USA). The value of *p* < 0.05 was considered significant between different symbols.

## Results

### Phy shows cytotoxicity in MCF-7 cells via mitochondrial apoptosis

**Figure 1.**
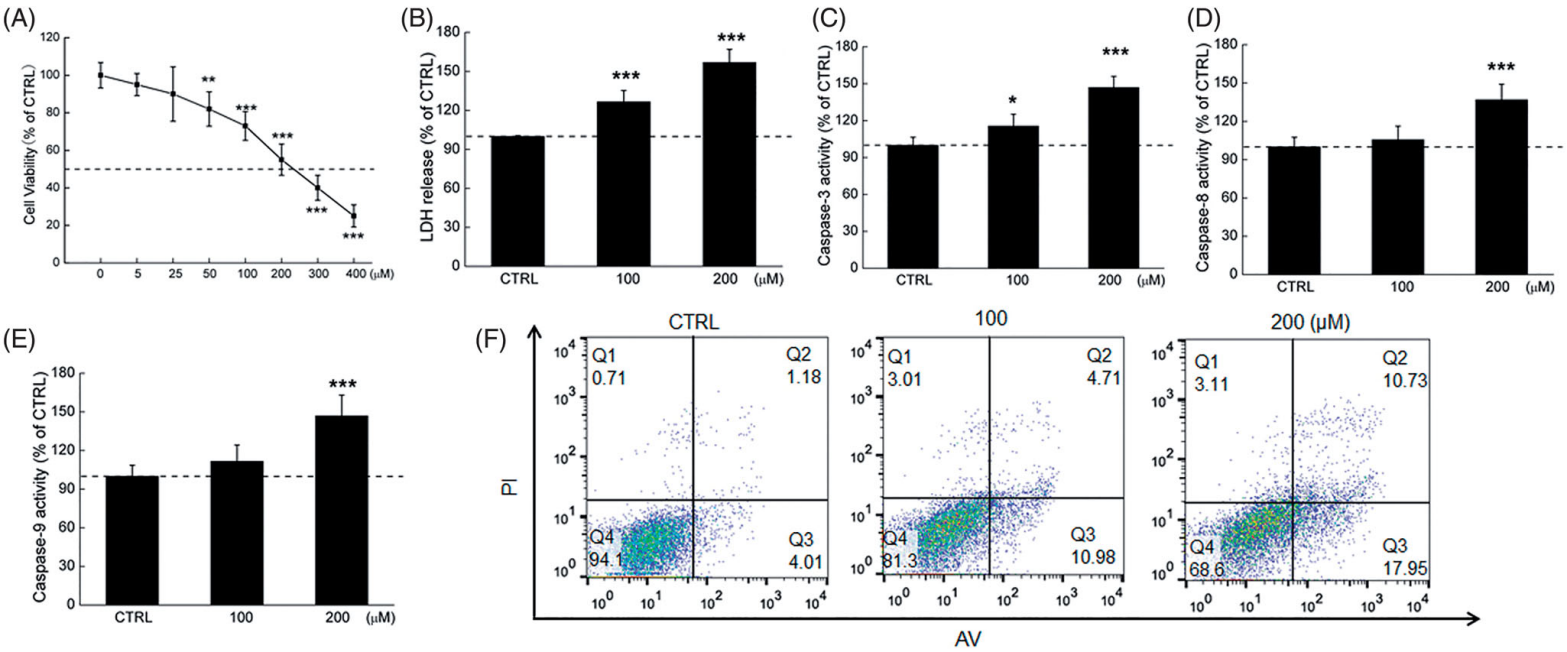
The cytotoxicity of Phy in breast cancer cells. (A) Phy suppressed the cell viability of MCF-7 cells measured by MTT assay after 24 h exposure. (B) Phy enhanced the release of LDH in MCF-7 cells. Twenty-four hour Phy incubation enhanced the activities of (C) caspase-3, (D) caspase-8, and (E) caspase-9 in MCF-7 cells. (F) Phy enhanced the apoptosis rate of MCF-7 cells after 24 h exposure. Data are expressed as percentages relative to the corresponding control cells and as mean ± S.D. (*n* = 6). *p<0.05, **p<0.01, and ***p<0.001 versus control cells.

**Figure 2.**
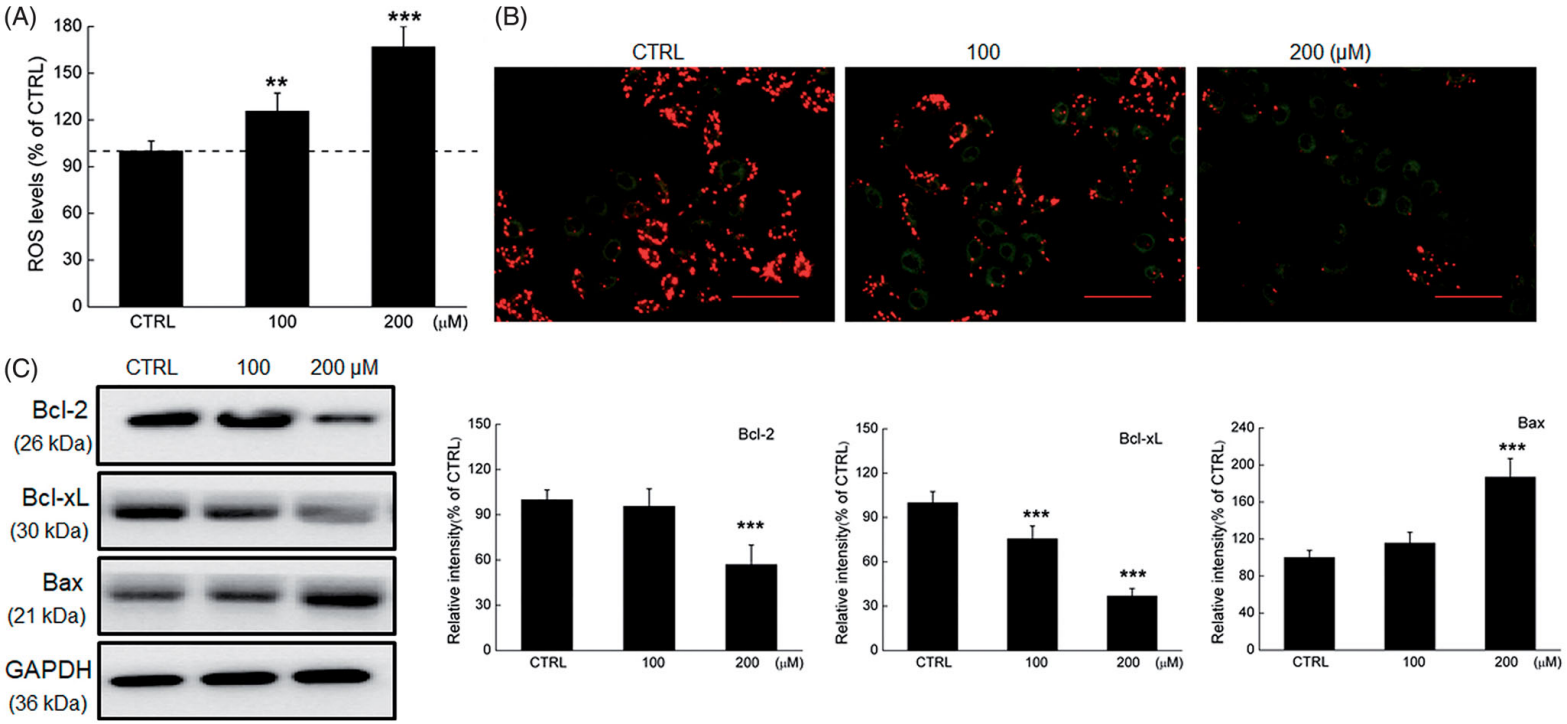
Phy caused mitochondrial dysfunction in breast cancer cells. Phy (A) upregulated ROS production and (B) decreased the MMP (20× magnification; scale bar: 100 μm) in MCF-7 cells after 24 h treatment. (C) Phy distinctly reduced the expression levels of Bcl-2 and Bcl-xL, and enhanced the expression levels of Bax in MCF-7 cells. Quantitative protein expression data were normalised to GAPDH levels in the corresponding samples. Data are expressed as percentages relative to the corresponding control cells and mean ± S.D. (*n* = 6). **p<0.01 and ***p<0.001 versus control cells.

Phy 24 h treatment significantly suppressed the MCF-7 cell viability at dose of 50–400 µM (*p* < 0.01) (Figure 1(A)), and the 24 h IC_50_ in MCF-7 cells is approximately 203.1 µM. The significant increases of LDH release as well as enhancements of caspase-3, -8, -9 activities, serving as critical participants in intrinsic and extrinsic mitochondrial apoptoic signalling, were found by Phy-treated cells (*p* < 0.001 at 200 µM, Figure 1(B–E)) (Hengartner 2000). Phy induced 56.9% (*p* < 0.001), 46.9% (*p* < 0.05), 36.9% (*p* < 0.001) and 46.9% (*p* < 0.001) increment on LDH release (Figure 1(B)), and the activities of caspase-3 (Figure 1(C)), caspase-8 (Figure 1(D)) and caspase-9 (Figure 1(E)) compared with CTRL cells. Phy, especially at 200 µM, caused 28.68% of early/late apoptosis in MCF-7 cells compared with non-treated cells (Figure 2(F)).

Phy caused the over-accumulation of intracellular ROS (*p* < 0.01) (Figure 2(A)), which induces apoptosis or even necrosis through oxidative stress of MCF-7 cells (Circu and Aw 2010). Twenty-four h exposure of Phy significantly caused the dissipation of MMP supporting by the reduced red to green fluorescence ratio (Figure 2(B)).

Bcl-2 family members initiate or inhibit apoptosis through regulating the mitochondrial permeability in the nucleus (Shimizu et al. 1999). Twenty-four h Phy incubation, especially at 200 µM, obviously reduced the expression levels of Bcl-2 and Bcl-xL, and promoted the expression levels of Bax in MCF-7 cells compared with the control cells (*p* < 0.001) (Figure 2(C)).

### Phy suppresses MCF-7-xenografted tumour growth in BALB/c nude mice relating to oxidative stress-mediated mitochondrial apoptosis

**Figure 3.**
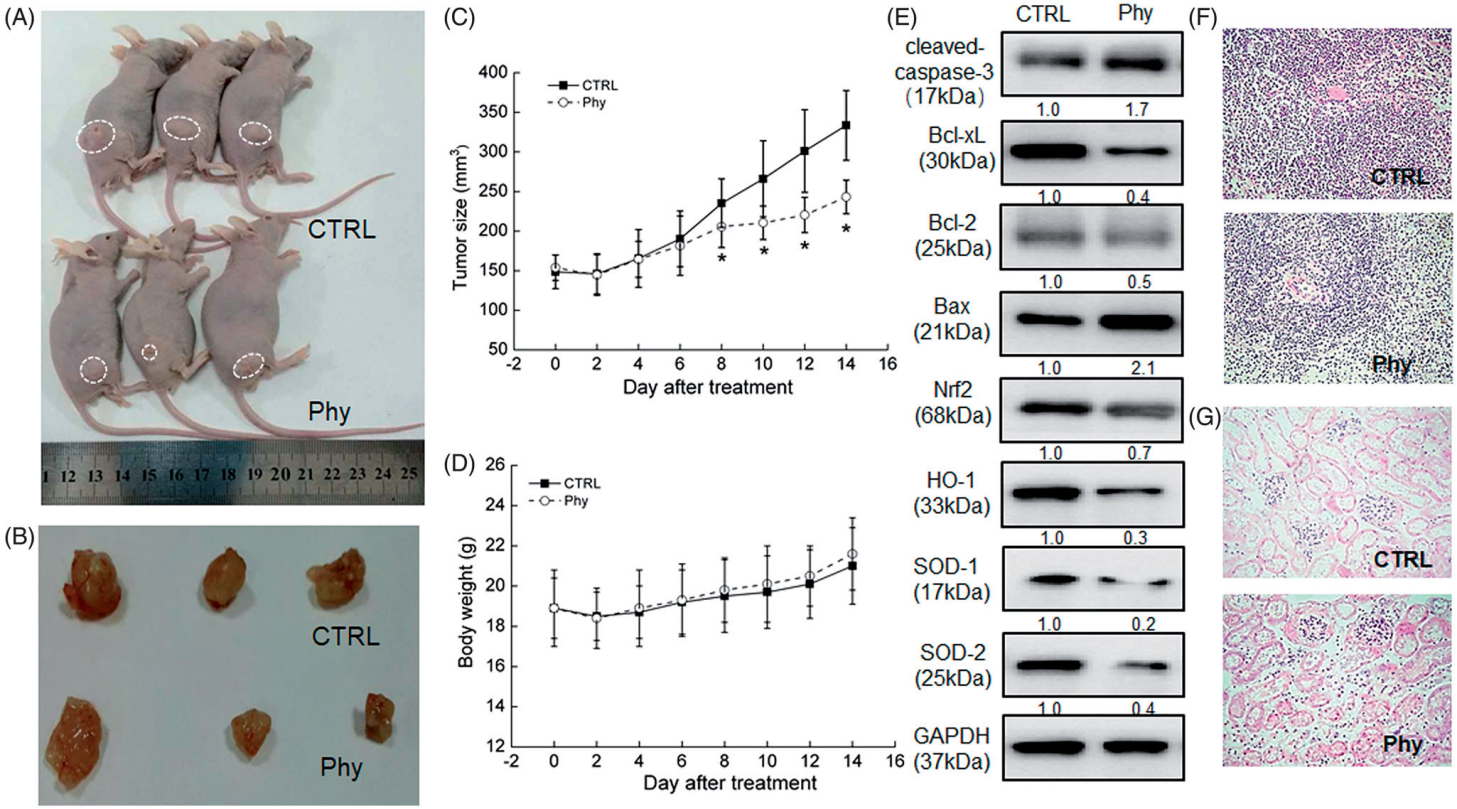
Phy suppressed MCF-7-xenograft tumour growth in BALB/c nude mice. MCF-7-xenograft BALB/c nude mice model were treated with Phy at 30 mg/kg every other day for 14 days. (A) Tumour-bearing nude mice. (B) Tumour tissue specimens. (C) Tumour sizes of MCF-7-xenografted nude mice in the control and Phy-treated groups. Tumour sizes are expressed as mean ± S.D. (*n* = 3). *p<0.05 versus control group. (D) Mean (± S.D.) body weight of the control and Phy-treated mice (*n* = 3). (E) Phy enhanced the expression levels of Bax and cleaved caspase-3, and reduced the expression levels of Bcl-2, Bcl-xL, Nrf2 and its downstream proteins. Quantitative protein expression data were normalised to the corresponding GAPDH levels, and the average fold changes in band intensity are marked (*n* = 3). Haematoxylin and eosin staining of liver (F) and spleen (G) tissues from nude mice.

MCF-7-xenografted BALB/c nude mice intraperitoneally injected with 30 mg/kg of Phy for 14 days were applied to investigate its anti-breast cancer activity. The tumour growth was significantly suppressed by Phy apparently from the 8th day compared with that of observed in CTRL group (*p* < 0.05) (Figure 3(A–C)) without influenced their bodyweights (Figure 3(D)) and organ structures including liver and spleen (Figure 3(F,G)), suggesting its safety for mouse treatment.

In tumour tissues of Phy-treated nude mice, Phy sharply enhanced the expression levels of cleaved-caspase-3 and Bax, and suppressed the expression levels of Bcl-2 and Bcl-xL (Figure 3(E)). Furthermore, Phy strongly suppressed the expression levels of Nrf2 and its downstream proteins including HO-1, SOD-1 and SOD-2 in the tumour lysis of MCF-7-xenografted in BALB/c nude mice (Figure 3(E)).

### Phy suppresses MCF-7-xenografted tumour growth in BALB/c mice relating to oxidative stress-mediated immunoregulation

**Figure 4.**
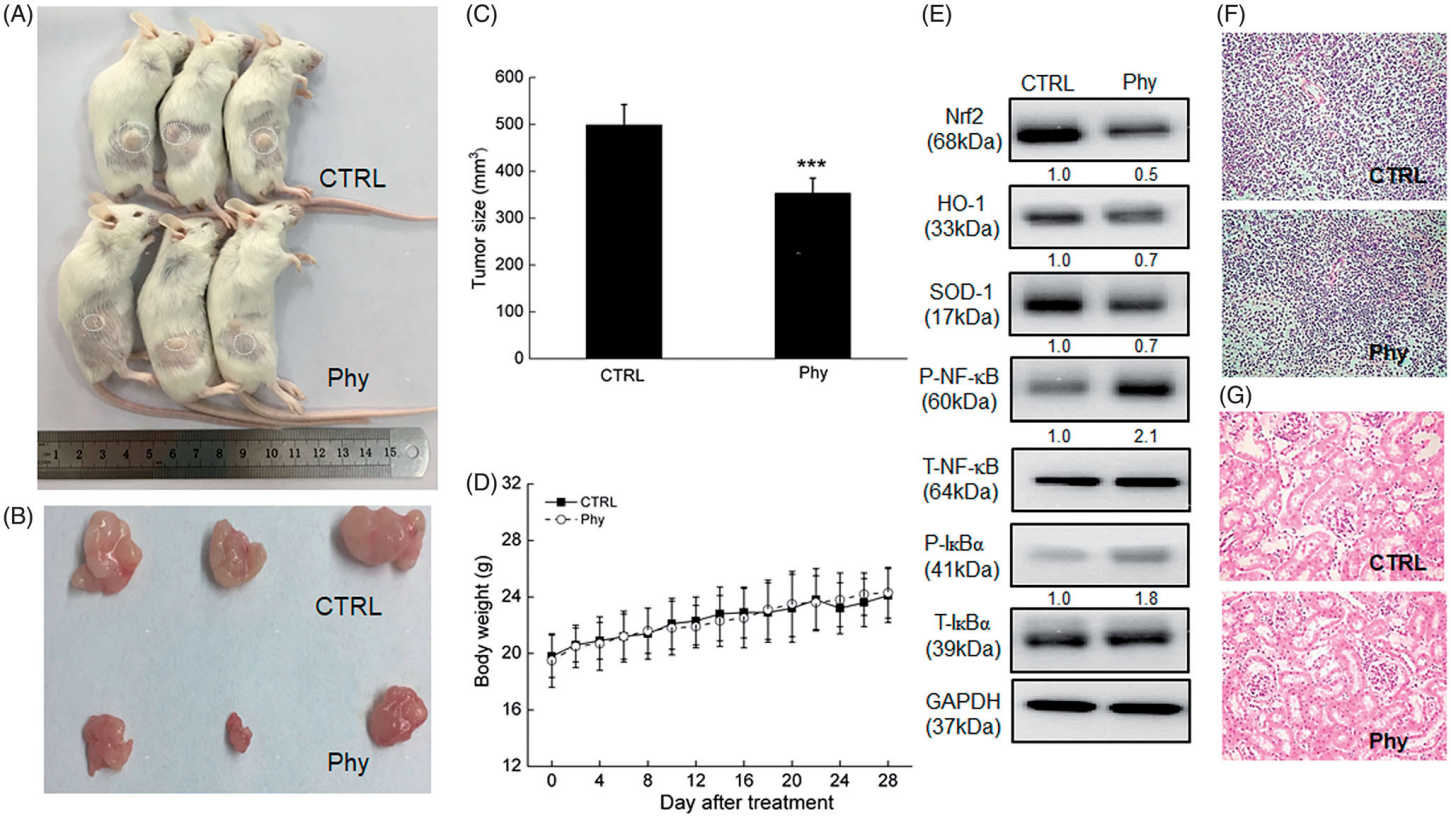
Phy suppressed MCF-7-xenograft tumour growth in BALB/c mice. The BALB/c mouse model was treated with Phy at 30 mg/kg every other day for 28 days. (A) Tumour-bearing nude mice. (B) Tumour tissue specimens. (C) Tumour sizes of MCF-7-xenografted mice in the control and Phy-treated groups. Tumour sizes are expressed as mean ± S.D. (*n* = 3). ***p < 0.001 versus control group. (D) Mean (± S.D.) body weight of the control and Phy-treated mice (*n* = 3). (E) Phy enhanced the phosphorylation of IκBα and NF-κB, and reduced the expression levels of Nrf2 and its downstream proteins. Quantitative protein expression data were normalized to the corresponding GAPDH levels, and the average fold changes in band intensity are marked (*n* = 3). Haematoxylin and eosin staining of liver (F) and spleen (G) tissues from nude mice.

In MCF-7-xenografted BALB/c mice, 28-day Phy injection evidently suppressed the tumour growth compared with CTRL group (342 vs. 501 mm^3^) (*p* < 0.001) (Figure 4(A–C)) without influencing their body weights (Figure 4(D)) and organ structures including liver and spleen (Figure 4(F,G)).

In serum of MCF-7-xenografted BALB/c mice, Phy injection resulted in 37.6, 19.8, 29.5, 18.9, and 18.9% increment on the levels of TNF-α (*p<*0.01), TNF-β (*p<*0.05), IFN-γ (*p<*0.01), IL-1β (*p<*0.05) and IL-2 (*p<*0.05), and 19.9, 27.5, 18.4, and 21.5% reduction on the levels of IL-6 (*p<*0.05), IL-10 (*p<*0.01), MMP-2 (*p<*0.01) and MMP-9 (*p<*0.05) (Table 1).

**Table 1.** The effects of Phy on the immune factors in serum of MCF-7-xenografted BALB/C mice.

Factor (pg/ml)	CTRL	Phy
TNF-α	289.6 ± 22.5	398.5 ± 23.6**
TNF-β	201.9 ± 27.9	241.9 ± 20.6*
IFN-γ	302.7 ± 42.9	392.1 ± 50.6**
IL-1β	100.5 ± 12.2	119.5 ± 9.5*
IL-2	352.9 ± 40.6	419.6 ± 49.2*
IL-6	75.2 ± 8.3	60.2 ± 7.5*
IL-10	69.2 ± 9.8	50.2 ± 4.9**
MMP-2 (*10^3^)	121.6 ± 12.3	99.2 ± 8.9**
MMP-9 (*10^3^)	53.9 ± 6.7	42.3 ± 5.4*

Results are represented as means ± S.D. (*n* = 3). Relative to the control group, *p < 0.05 and **p < 0.01.

In MCF-7-xenografted BALB/c mice, Phy regulated the oxidative stress, evidencing by reducing the serum ROS levels (*p<*0.01), and enhancing the serum SOD (*p<*0.01) and CAT levels (*p* < 0.05) (Table 2).

**Table 2.** The effects of Phy on oxidative factors of serum in tumour- xenografted BALB/c mice.

Factor (U/ml)	CTRL	Phy
ROS	216.3 ± 24.1	156.2 ± 18.5**
SOD	78.2 ± 11.6	123.6 ± 9.9**
CAT	74.1 ± 9.6	98.3 ± 8.9*

Results are represented as means ± SD (*n* = 3). Relative to the control group, *p < 0.05, and **p < 0.01.

In the tumour tissues of MCF-7-xenografted BALB/c mice, Phy strongly suppressed the expression levels of Nrf2, HO-1 and SOD-1 (Figure 4(E)). Additionally, Phy increased the phosphorylation levels of IκBα and NF-κB in tumour tissue of MCF-7-xenografted tumour BALB/c mice (Figure 4(E)).

## Discussion

The safety of green natural products has raised concerns among the public. In this study, Phy has been confirmed to show anti-breast cancer properties in MCF-7 cells and its xenografted nude mice and immunosuppressive mice via modulation the mitochondrial apoptosis and immune response, especially via its regulation on oxidative stress. Encouragingly, Phy failed to influence the bodyweight and organs (liver and spleen) structures in both the nude mice and immunosuppressive normal BALB/c mice, which suggests its medication safety in trials.

During the pro-apoptotic process on breast cancer cells, Phy significantly enhanced cell apoptosis and activities of caspases, induced the dissipation of MMP and the over-accumulation of intracellular ROS, which is responsible for the loss of function in the mitochondria. As the energy metabolism centre of eukaryotic cells, mitochondria is involved in cell transmission and apoptosis (Cao et al. 2011). Numerous studies demonstrate that the overproduction of ROS and the abnormal expressions of Bcl-2 family members contribute to mitochondrial permeability transition (MPT) which is considered as the index of mitochondrial apoptosis (Wang and Youle 2009). Following this process, the pro-apoptotic molecules releasing from the mitochondria, which help to activate the caspase family members and other catabolic enzymes (Desagher and Martinou 2000). Intracellular ROS serving as a second messenger of extracellular growth factors to influence the cytokines and/or hormones causing the mitochondrial oxidative damage and apoptosis via Nrf2 signalling, which consequently leads to further ROS leakage from the mitochondria (Ivanov et al. 2011; Chao et al. 2017; Li H et al. 2018).

Phy distinctly increased the expression levels of Bax, and decreased the expression levels of Bcl-xL and Bcl-2. The formation of Bcl-2/Bax heterodimer exhibits pro-apoptotic effect via blocking Bcl-2 which contributes to the generation of ROS and the regulation on the MMP levels (Noujaim et al. 2002; Chong et al. 2014; Alarifi et al. 2017). A feedback amplification loop drives the generation of ROS in mitochondria. The overproduction of ROS activates caspase-8, which indirectly activates caspase-9; consequently, caspase-3, for the execution of the apoptotic program, is activated (Budihardjo et al. 1999; Kim and Chung 2007).

Besides, the reduction of antioxidant factors including Nrf2, HO-1 and SOD in tumour tissues induced by Phy further contributes to the ROS accumulation. Nrf2 is highly expressed in most human tumours, and is related to the expression of various oncogenes. Considerable researches of antitumor drugs demonstrate that blocking the Nrf2 signalling pathway can reduce tumour recurrence rates and increase tumour sensitivity to radiotherapy and chemotherapy drugs (Cong et al. 2014). All these findings suggest that oxidative stress-mediated mitochondrial apoptosis, at least partially, is involved in the cytotoxicity of Phy in breast cancer cells.

Inflammation and immune response participate in the tumour development of initiation, promotion, malignant conversion, and metastasis (Singh et al. 2017). In MCF-7-xenografted BALB/c mice, Phy modulated the serum levels of ILs, TNFs, IFN-γ and MMPs, which has been reported to show important roles in the inflammatory process of lesions and malignancies of breast cancer. In tumour patients, inflammation is mainly mediated by inflammatory cells, immune cells, various inflammatory factors and their regulatory networks (Kamate et al. 2002; Kraus and Arber 2009; Calabrese et al. 2018). The reduction of IL-6 and IL-10 recognized as pleiotropic immunomodulatory factor, contribute to immune regulation and tumour angiogenesis (Zdravkovic et al. 2014). MMPs are involved in the process of cancer development. Among them, MMP-2 and MMP-9 are two important enzymes that directly lead to tumour metastasis and neovascularization (Pepper 2001).

NF-κB, a transcription factor of Rel family, participates in cell physiological activities through regulating gene transcription related to immunoregulation and inflammatory. According to previous research, the inhibitory protein IκBα can be phosphorylated by the enzyme IκB kinase (IKK), and then dissociated from NF-κB in the cytosol, finally leading to the phosphorylated activation of NF-κB (Philip and Kundu 2003). In MCF-7-xenografted tumour of ALB/c mice, Phy enhanced the phosphorylation levels of NF-κB via promoting the phosphorylation of IκBα, thus inhibiting the secretion of immunoregulatory factors and inflammatory factors. NF-κB is also a major activating factor in the inflammatory response which has a higher expression level in most malignant tumour cells (Martins et al. 2009; Tao et al. 2016). After NF-kB activation, it regulates the transcription of ILs and some growth factors, which initiate unstoppable proliferation of peripheral inflammation-related cells via endocrine or paracrine pathway (Konturek et al. 2004; Tabata et al. 2014). In TNF-induced apoptosis and inflammatory responses, ROS acts as an important second messenger in the TNF signalling pathway and is involved in regulating the transcriptional activity of NF-κB (Bohler et al. 2000; Ma et al. 2015). Natural compounds can show dual functions of anti-inflammatory, immunomodulation and anti-oxidation via modulating Nrf2/NF-κB signalling (Li W et al. 2008; Surh 2008; Calabrese et al. 2010; Das et al. 2012). Pharmacological and genetic studies suggest the functional cross-talk between Nrf2 and NFκB signalling pathways. The reduction of Nrf2 can enhance NF-κB activity which leads to increased cytokine production, whereas NF-κB modulates Nrf2 transcription and activity via regulating the target gene expression (Wardyn et al. 2015).

Studies have shown that anthraquinone and polyphenols can enhance the activity of antioxidant enzymes to scavenge free radicals, perform anti-inflammatory effect, inhibit apoptosis in cerebral ischaemia, and prevent as well as treat aging-related degenerative diseases (Li et al. 2019; Brunetti et al. 2020; Di Rosa et al. 2020; Leri et al. 2020). Physcion as an anthraquinone substance has been proven to enhance the activity of nerve cells, reduce the expression of lactate dehydrogenase (LDH) and c-fos protein, and enhance the activity of antioxidant enzymes, thereby reducing the neuronal damage caused by hypoxia (Pang et al. 2016; Li et al. 2019). Our study proved that physcion can regulate the level of oxidative stress in breast cancer cells, and affect the expression of inflammatory factors, which contributes to the apoptosis of breast cancer cell. In the future research, the safety and the applicable dose of physcion as a breast cancer treatment agent in human, meanwhile, the possible adverse reactions of the human body still need further research. In addition, in view of the protective effect of physcion on nerve cells, the application of physcion in the treatment or adjuvant treatment of brain tumours is an area worthy of attention.

## Conclusions

This research demonstrated the anti-breast cancer property of Phy in cells and two tumour xenografted mice models via regulation the oxidative stress-mediated mitochondrial apoptosis and immune response, especially related to Nrf2/NF-κB signalling. These findings provide pharmacological support for Phy as a candidate drug therapy for breast cancer.
